# Bayesian biclustering of gene expression data

**DOI:** 10.1186/1471-2164-9-S1-S4

**Published:** 2008-03-20

**Authors:** Jiajun Gu, Jun S Liu

**Affiliations:** 1 School of Engineering and Applied Sciences, Harvard University, Cambridge, Massachusetts, USA; 2 Department of Statistics, Harvard University, Cambridge, Massachusetts, USA

## Abstract

**Background:**

Biclustering of gene expression data searches for local patterns of gene expression. A bicluster (or a two-way cluster) is defined as a set of genes whose expression profiles are mutually similar within a subset of experimental conditions/samples. Although several biclustering algorithms have been studied, few are based on rigorous statistical models.

**Results:**

We developed a Bayesian biclustering model (BBC), and implemented a Gibbs sampling procedure for its statistical inference. We showed that Bayesian biclustering model can correctly identify multiple clusters of gene expression data. Using simulated data both from the model and with realistic characters, we demonstrated the BBC algorithm outperforms other methods in both robustness and accuracy. We also showed that the model is stable for two normalization methods, the interquartile range normalization and the smallest quartile range normalization. Applying the BBC algorithm to the yeast expression data, we observed that majority of the biclusters we found are supported by significant biological evidences, such as enrichments of gene functions and transcription factor binding sites in the corresponding promoter sequences.

**Conclusions:**

The BBC algorithm is shown to be a robust model-based biclustering method that can discover biologically significant gene-condition clusters in microarray data. The BBC model can easily handle missing data via Monte Carlo imputation and has the potential to be extended to integrated study of gene transcription networks.

## Background

Clustering gene expression data has been an important problem in computational biology. While traditional clustering methods, such as hierarchical and K-means clustering, have been shown useful in analyzing microarray data, they have some limitations. First, a gene or an experimental condition can be assigned to only one cluster. Second, all genes and conditions have to be assigned to clusters. However, biologically a gene or a sample could participate in multiple biological pathways, and a cellular process is generally active only under a subset of genes or experimental conditions. A biclustering scheme that produces gene and condition/sample clusters simultaneously can model the situation where a gene or a condition is involved in several biological functions. Furthermore, a biclustering model can avoid those “noise” genes that are not active in any experimental condition.

Biclustering of microarray data was first introduced by Cheng and Church [1]. They defined a residual score to search for submatrices as biclusters. This is a heuristic method and can not model the cases where two biclusters overlap with each other. Segal et al. [2] proposed a modified version of one-way clustering using a Bayesian model in which genes can belong to multiple clusters or none of the clusters. But it can not simultaneously cluster conditions/samples. Tseng and Wong developed a tight clustering algorithm [3]. It allows some of the genes not to be clustered, but does not select conditions. Bergmann et al [4] introduced the iterative signature algorithm (ISA), which searches bicluster modules iteratively based on two pre-determined thresholds. ISA can identify multiple biclusters, but is highly sensitive to the threshold values and tends to select a strong bicluster many times. The plaid model [5] introduces a statistical model assuming that the expression value in a bicluster is the sum of the main effect, the gene effect, the condition effect, and the noise term, i.e.:

$$y_{ij}=\mu +\alpha_{i}+\beta_{j}+\varepsilon_{ij},$$

where noise ε*_ij_* ~ *N*(0, σ^2^). It further assumes that the expression values of two overlapping biclusters are the sum of the two module effects. The plaid model uses a greedy search strategy, so errors can accumulate easily. Also in multiple clusters case, the clusters identified by the algorithm tend to overlap to a great extent. Tanay et al. [6] proposed a SAMBA biclustering scheme using bipartite graphs containing both conditions and genes. Ben-Dor et al. [7] attempted to identify order-preserving sub matrices (OPSMs). Murali and Kasif [8] discretized gene expression data into several symbols and searched for conservative symbol motifs (xMOTIFs). A survey of different biclustering methods can be found in [9].

We here propose a Bayesian biclustering (BBC) model. For a single bicluster, we assume the same model as in the plaid model [5], as described in equation (1). But for multiple clusters, we constrain the overlapping of biclusters to only one direction (i.e., either gene or condition direction). Besides, we use a more flexible error model, which allows the error term of each cluster to have to a different variance. To make the Bayesian inference of biclusters, we implemented an efficient Gibbs sampling algorithm with all effect parameters (except the error variances) integrated out. We compared the performance of the BBC algorithm for several different types of simulated datasets with that of the plaid model [5], the ISA [4], the method of Cheng and Church [1], the SAMBA method [6] and the OPSMs [7]. Finally, we applied the BBC algorithm to the yeast expression dataset and identified many biologically significant biclusters.

## Results and discussion

### Simulation results

#### Bayesian biclustering in various simulated scenarios

We simulated a dataset with 400 genes and 50 samples. The background data is i.i.d. from *N*(0, 0.5). Two clusters of 100 genes and 15 conditions are simulated according to the BBC model with main effects, gene effects, condition effects and error terms as *μ*_1_ ~ *N*(5, 0.5), *μ*_2_ ~ *N*(7, 0.5), α_*i*1_, α_*i*2_ ~ *N*(0, 0.5), β_*j*1_, β_*j*2_ ~ *N*(0, 0.5) and ε_*ij*1_ ~ *N*(0, 0.5), ε_*ij*2_ ~ *N*(0, 0.7).

**Figure 1:**
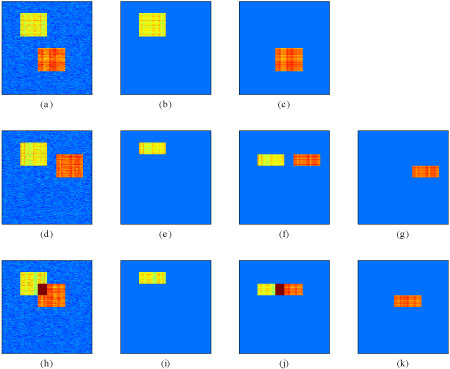
**Simulated data with two biclusters and the results of the BBC analysis.** Bayesian biclustering for simulated datasets. (a) A dataset with two non-overlapping clusters. (b)-(c) The two clusters found by the Bayesian biclustering model from (a). (d) A dataset with two clusters with common genes. (e)-(g) The three clusters found by the Bayesian biclustering model from (d). (h) A dataset with two clusters with both common samples and common genes. (i)-(k) The three clusters found by the Bayesian biclustering model from (h).

We considered three scenarios for datasets with two clusters: the two clusters have some common conditions but distinct genes (Figure 1(a)); the two clusters have some common genes but distinct conditions ( Figure 1(d)); and two clusters have both common genes and conditions (Figure 1(h)), in which case an additive model is assumed for the overlapping part. The results from using a non-overlapping gene version of the BBC model are shown in Figures 1(b)-(c),(e)-(g),(i)-(k). In all cases the BBC model identified the genes and conditions of the simulated clusters correctly, but grouped them slightly differently because of our model constraints.

#### Comparison of biclustering algorithms on data simulated from statistical models

**Figure 2:**
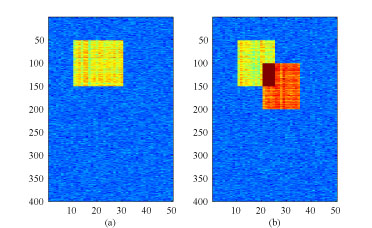
**Datasets simulated according to the plaid model** Datasets for comparison. (a) A dataset with one single cluster (b) A dataset with two clusters, of which both genes and samples overlap.

We compared six biclustering methods: the BBC method, the plaid model, ISA, SAMBA, OPSMs, and Cheng and Church's biclustering (CC). We considered both the single cluster case and the multiple clusters case using simulated data from the plaid model. A single cluster dataset is shown in Figure 2(a). The 400 × 50 background noise matrix is simulated according to i.i.d. normal *N*(0,0.5). We superimposed a cluster of size 100 × 20 according to the plaid model with μ_1_ ~ *N*(5, 0.5) and α_*i*1_, β_*j*1_ ~ *N*(0, 0.5). The multiple cluster case is shown in Figure 2(b). The background is the same as above. Two clusters of size 100 × 15 are also simulated according to the plaid model with μ_1_ ~ *N*(5,0.5), μ_2_ ~ *N*(7, 0.5) and α_*i*1_, β_*ji*_, α_*i2*_, β_*j2*_ ~ *N*(0, 0.5). An additive model is used for the overlapping part of the two clusters.

Since each method searches for biclusters with different structures, comparing biclustering results is not very straightforward. In order to carry out a comprehensive comparison among various biclustering results for simulated datasets, we use the following four characteristics: sensitivity, specificity, overlapping rate, and number of clusters. Since we know which gene-condition combination belongs to the true biclusters, we use the standard definition for sensitivity and specificity, both of which are values between 0 and 1. A higher sensitivity suggests that more “true” members of the clusters have been identified by the algorithm, while a higher specificity suggests that more background data points are excluded from the clusters. The overlapping rate is defined as

$$1-\frac{\#ofmatrixentriesintheunionofidentifiedclusters}{\sum_{allclusters}\#ofentriesineachidentifiedcluster}$$

Thus, if there is no overlap between the identified clusters, the overlapping rate is 0. On the other hand, if the identified clusters greatly overlap with each other, the overlapping rate is close to 1.

We used the BicAT software package [10] for ISA, CC, and OPSMs. Different gene and condition thresholds are used for the ISA. We carefully chose a set of thresholds with good performance and then slightly changed the thresholds to test the stability of the ISA. We used default settings for CC's model. The plaid algorithm was implemented using the Plaid package [5]. The Expander package [11] is used for SAMBA biclustering. The results are shown in Table 1, where the left-hand-side value in each entry is for the single cluster case, and the right-hand-side value is for the two-clusters case.

**Table 1:** Biclustering results of different methods for simulated data using the plaid model

	Sensitivity		Specificity		Overlapping rate		# of clusters	
ISA (0.6, 1)	1	0.84	0.99	0.84	0	0.12	1	3
ISA (0.6, 1.2)	0.95	0.53	0.84	0.90	0.06	0.08	10	8
ISA (0.7, 1.1)	0.84	0.68	0.91	0.84	0	0.16	10	8
SAMBA	0.43	0.39	0.99	0.99	0.31	0.3	7	14
CC*	1	0.98	0	0	0.02	0	10	10
OPSMs	0.38	0.25	0.94	0.96	0.3	0.5	11	12
Plaid	1	1	1	0.73	0	0.63	1	11
BBC**	1	1	1	1	0	0	1	3

Note: *In CC's method, the number of clusters is preset to be 10. **In BBC, the overlapping rate is automatically 0.

It can be seen that the ISA method is very sensitive to the choice of the thresholds. The performance of ISA also degrades in the case of multiple overlapping clusters. The SAMBA method and the OPSMs method correctly identified almost all background noises, but tends to exclude some meaningful patterns. The CC method includes too much background data in clusters. The plaid model performs well in the single cluster case. But it identifies too many overlapped clusters in the multiple clusters case. Our BBC method performs well in both cases, even though the data generation model for the overlapping part in the second case does not satisfy the BBC model assumption.

#### Comparison of biclustering algorithms on data simulated with biological characteristics

**Figure 3:**
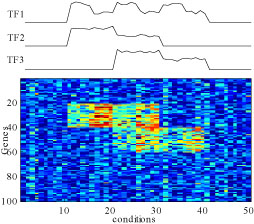
The Simulated dataset with realistic characters

People are mostly interested in how different biclustering methods perform for real microarray datasets. We next carry out a comparison using simulated microarray datasets with realistic characteristics. As shown in Figure 3, the dataset has 100 genes and 50 conditions. The concentration of three transcription factors (TF) are changed across conditions. We assume that gene 21 to gene 40 are transcribed when both TF1 and TF2 are active, with TF1 serving as an activator and TF2 as an inhibitor. We also assume that gene 41 to gene 60 are transcribed when both TF1 and TF3 are active, where TF1 serves as an inhibitor, TF3 an activator. The expression values are simulated using the biochemistry model presented in [12]. If gene *i* is regulated by *M_I_* inhibitors and *M_A_* activators, then

$$\frac{dG_{i}}{dt}=s(I_{1},I_{2}\ldots ,I_{M_{I}},A_{1},A_{2},\ldots A_{M_{A}})-b(G_{i})$$

where *G_i_* represents the abundance of the mRNA of gene *i*, *s*(*I*_1_, *I*_2_ …, *I_M_I__*, *A*_1_, *A*_2_, … *A_M_A__*) is a rate law representing mRNA synthesis, *I*_1_, *I*_2_ …, *I_M_I__* are inhibitor concentrations, *A*_1_, *A*_2_…, *A_M_A__* are activator concentrations, and *b(G_i_)* is mRNA breakdown rate. In [12] mRNA synthesis rate is modelled as

$$s(I_{1},I_{2}\ldots ,I_{M_{I}},A_{1},A_{2},\ldots A_{M_{A}})=V_{basal}\prod_{j}^{M_{I}}(\frac{Ki_{j}^{n_{j}}}{I_{j}^{n_{j}}+Ki_{j}^{n_{j}}})\prod_{k}^{M_{A}}\left(1+\frac{A_{k}^{n_{k}}}{A_{k}^{n_{k}}+Ka_{k}^{n_{k}}}\right)$$

where *V_basal_* is the basal transcription rate, constants *Ki_j_* and *Ka_k_* are concentrations at which the effect of the inhibitor or activator is half of its saturating value. The exponents *n_i_* and *n_k_* regulate the sigmoidicity of the transcription rate curve. We set *n_i_* = *n_k_* = 1.5, and randomly simulated *Ki_j_* and *Ka_k_* for the dataset.

We added real noise from the well known Leukemia expression dataset [13]. We first obtained a noise data matrix using all scattered (“noise”) genes excluded by the tight clustering algorithm [3]. Then, we chose 100 rows and 50 columns at random. We also scaled the noise to control signal to noise ratio (SNR). Both the good data quality case (SNR=10) and the bad data quality case (SNR=4) are considered. We simulated 10 datasets for both cases and the average value of each characteristics is shown in Table 2, where the left-hand-side value in each entry is for SNR=10 case, and the right-hand-side value is for SNR=4 case. we chose the threshold values for the ISA model in the same way as in the previous simulation study.

**Table 2:** Biclustering results of different methods for simulated data with realistic characteristics

	Sensitivity		Specificity		Overlapping rate		# of clusters	
ISA (0.6, 1)	0.98	0.70	0.76	0.78	0.51	0.65	7.2	9.8
ISA (0.6, 1.2)	0.90	0.75	0.79	0.73	0.57	0.57	11.2	13
ISA (0.7, 1.1)	0.94	0.76	0.80	0.79	0.48	0.59	8.3	10.9
SAMBA	0.38	0.28	0.99	0.99	0.37	0.37	5.8	5.3
CC*	0.84	0.70	0.15	0.25	0.02	0.01	10	10
OPSMs	0.21	0.16	0.91	0.91	0.35	0.35	9.3	8.9
Plaid	1.00	0.99	0.48	0.61	0.30	0.18	5	2.9
BBC**	1	0.97	0.99	0.97	0	0	2	2

Note: *In CC's method, the number of clusters is preset to be 10. **In BBC, the overlapping rate is automatically 0.

The BBC model performed the best among these methods. Again the ISA method was sensitive to thresholds. It also had some false positives. The OPSMs method missed most of the significant patterns. The SAMBA method found some small and tight biclusters of genes and conditions, but also excluded many significant patterns. CC's method misidentified many noisy data points as biclusters. The plaid model recognized almost all significant patterns, but its specificity was low. Interestingly, the plaid model gave better results for low SNR case, which was due to the fewer number of clusters found by the plaid model.

#### Effects of normalization for Bayesian biclustering model

Data normalization is an important step for microarray analysis. Although some clustering methods such as ISA incorporate the normalization step in their procedures, most clustering methods work on normalized microarray data. The BBC model belongs to the latter. Since the normalization procedure greatly changes the microarray data, different normalization procedures may lead to very different clustering results.

We conducted a study on how normalization methods affect the biclustering results. Five normalization procedures including column standardization (CSN), row standardization (RSN), quantile normalization on gene level (QNGL), the interquartile range normalization (IQRN), and the smallest quartile range normalization (SQRN) were considered. In CSN (or RSN), each column (or row) is re-centered and re-scaled, so that the sample mean of each column (or row) becomes 0, and the sample variance becomes 1. These are quite crude methods, but are still used in many clustering applications. QNGL used the same technique with quantile normalization [14], which was developed for normalizing oligonucleotide arrays on probe levels. In QNGL, we apply the quantile normalization on gene level for the simulated array data.

IQRN and SQRN are two new methods we propose here. They are inspired by CSN, but are more robust to outliers. In IQRN, one first sorts the data in each column, trims off α/2% of the data from each tail, and computes the α%-trimmed mean and standard deviation. Then, all data in that column is standardized by subtracting the trimmed mean and being divided by the trimmed standard deviation. This normalization method can reduce the artificial normalization effect caused by outliers. In SQRN, instead of using the middle (100-α)% of the data, one first finds for each column the shortest interval that contains a certain percentage (e.g., 50%) of the data. Then the data of that column is standardized by the sample mean and variance of the data inside the shortest quartile range. If distributions of the data in each column are symmetric and unimodal, then SQRN is equivalent to IQRN. But SQRN gives better results for skewed distributions. We applied the above five normalization methods on the same simulated dataset as in Figure 2(b) before applying our BBC procedure.

As shown in Table 3, both IQRN and SQRN performed very well, whereas the other three methods affected the clustering results significantly. Thus, in our yeast data analysis reported in the following section, we used IQRN before applying the BBC procedure.

**Table 3:** Comparison of normalization methods for Bayesian Biclustering Model

	Sensitivity	Specificity	Overlapping rate	# of clusters
RSN	0.84	0.85	0	3
CSN	0.95	0.58	0	3
QN	1	0.44	0	4
IQRN	1	1	0	3
SQRN	1	1	0	3

### Bayesian biclustering for yeast datasets

We analyzed the same yeast expression data as in [15] using the BBC procedure. This dataset was derived by combining the environmental stress data of [16] and the cell cycle data of [17]. The combined dataset contains 6108 genes and 250 conditions (or samples). We applied the 90% IQRN procedure across all conditions. Since the dataset contains many missing data, we imputed them along with our BBC iterations. The BBC algorithm was asked to search for *K* biclusters, with *K* ranging from 30 to 65. We observed that the BIC [18] achieved the optimal value with *K* = 57. Out of 6108 genes, 6021 were included in one of the clusters, and all conditions were included in at least one cluster.

We analyzed the clustering results from three aspects. First, we identified the significant categories of experimental conditions for each cluster. More precisely, we classified the 250 experimental conditions into 22 categories according to the biological nature of each experiment. Some examples of categories are heat shock stress, amino acid starvation, and *α* factor synchronization. Then we searched for significant enrichment for each category. Second, we did functional enrichment test of genes in each cluster using functional information from the MIPS database. Third, we searched the promoter sequences (up to 800 bps upstream) of genes in each bicluster for the enrichment of transcription factor binding sites (TFBS). We applied the TFBS scores of 51 known yeast transcription factors used in [15], which measures how likely a promoter sequence contains a TFBS and was computed using ScanACE [19]. A cutoff value of 0.5 was used to make a presence/absence call for a TFBS. The presence frequency of each TFBS in all genes in the dataset was used as null hypothesis. For all the three types of enrichment analysis, we used the criterion of P value< 0.05 after the Bonferroni correction.

Out of the 57 clusters, 36 have significant gene functions enrichments, 26 have significant TFBS enrichments, 51 have significant experimental condition categories enrichments, 22 have all three types of enrichments and 54 have at least one type of enrichment. We named a few of these biclusters and listed them in Table 4. The enriched gene functions and the significant experimental conditions showed strong correlations. For example, in the cell cycle cluster, the enriched gene function terms are cell cycle, DNA processing and cell division, and the significant experimental conditions are all cell-cycle experiments including cln3 and clb2 experiments, *α* factor, cdc15, cdc28 and elutriation synchronization. Another example is stress response and protein folding cluster, the enriched gene function terms are stress response and protein folding, and the significant conditions are heat shock, diamide and osmolarity stress experiments. The biclustering results are also supported by the TFBS information. In many clusters, the enriched TFBSs correspond to TFs known to be involved in the pathways and biological functions that were found significant from gene functional enrichment analysis. For instance, in the ribosome protein bicluster, the enriched TFBS RAP1 is known to be involved in ribosome protein transcription [20]. The ubiquitin cluster is enriched with TFBS of RPN4, which was shown to be part of the ubiquitin fusion degradation pathway [21]. The nitrogen, sulfur and selenium cluster shows significant over-representation of binding sites for TFs CBF1, GCN4, MET31, and MET4. CBF1 is known to induce sulfate assimilation pathway along with MET4 [22], GCN4 is an activator involved with protein biosynthesis, and MET31 is a known transcriptional regulator of sulfur amino acid metabolism [23]. The G1 phase cluster is enriched with MBP1 and SWI4 binding sites. MBP1 and SWI4 are known to act together to regulate late G1-specific transcription of targets and genes for DNA synthesis [24]. The oxidative stress cluster is enriched by CAD1 and YAP1 binding sites, where YAP1 activates the transcription of anti-oxidant genes in response to oxidative stress [25]. The glycolysis regulation cluster is enriched by TFBS GCR1, which is known to be involved in glycolysis [26].

**Table 4:** Bayesian Biclustering results for yeast expression data

Cluster name	size*	Significant conditions (P value)	Enriched TFBS (P value)	Enriched gene functions (P-value)
ribosome proteins	213,85	nitrogen depletion(7.1e-3), steady state (3.9e-4)	RAP1 (2.9e-60)	ribosomal protein (2.1e-160)
rRNA processing	329,113	steady state (8.9e-4)	ABF1 (5.2e-4), PAC (1.2e-127), RRPE (2.7e-63)	rRNA processing (4.3e-77), nucleic acid binding (1.6e-25)
ubiquitin	113,88	diamide stress(4.2e-3), menadione stress(2.7e-2)	RPN4 (4e-12)	ubiquitin / proteasomal pathway (8.3e-12)
oxidative stress	40,38	hydrogen peroxide stress (4.8e-8), menadione stress(4e-7), diamide stress (3.2e-6)	CAD1(5.7e-15), YAP1(1.9e-15)	oxidative stress response (9.3e-8), metabolism of phenylalanine (4.2e-8), metabolism of tyrosine (2.7e-8)
respiration	55,97	steady state(1.8e-7)	HAP4 (1.3e-16), SKN7(6.3e-8), MSN24a(7.4e-4)	respiration (2.5e-38), electron transport and membrane-associated energy conservation (5.1e-45)
purin metabolism	42,48	menadione stress (4.1e-6), amino acid starvation (4.8e-3)	BAS1 (3.2e-5)	purin nucleotide/nucleoside/nucleobase anabolism (6.2e-10)
stress response and protein folding	48,46	heat shock (4.5e-7), diamide stress (1.7e-4), osmolarity stress (6.5e-4) , MSN2/4 and YAP1 deletion (3.8e-3)	HSF1 (4.7e-3),	protein folding and stabilization (8e-8), stress response(3.0e-5)
stress response and heat shock	87,191	heat shock (5.2e-3)	HSF1 (1.5e-3), MSN24 (6.1e-11), MSN24a (9.6e-11), STRE (1.0e-5), GIS1 (1.9e-4)	C-compound and carbohydrate metabolism (1.0e-3), energy (7.4e-4)
cell cycle	86,87	α factor (3.5e-8), cdc15 (3.7e-8), cdc28 (4.5e-2), elu (4.0e-6)	MCM1 (1.0e-10), SWI4 (4.16e-7), FKH1 (6.6e-7), MBP1(3.6e-4), TATA (1.3e-4)	cell cycle and DNA processing (5.1e-9), cytokinesis (cell division) (2.9e-6), pheromone response (7.6e-4)
DNA topology	35,45	cln3, clb2 (2.1e-2)	GCN4(4.3e-6), MBP1 (2.0e-5), MCM1 (3.2e-3), SWI4 (1.1e-3), XBP1 (1.3e-5)	DNA topology (1.3e-22), somatic/ mitotic recombination (8.9e-9)
cell cycle (G1 phase)	108,62	α factor (3.35e-11), cdc 15 (2.5e-10), cdc28 (7.8e-6)	MBP1 (3.7e-14), SWI4 (6.4e-5)	cell cycle and DNA processing (1.4e-12)
nitrogen, sulfur & selenium metabolism	37,16	amino acid starvation (1.2e-5), nitrogen depletion (4.2e-2)	CBF1(3.3e-7), GCN4 (7.3e-5), MET31 (8.7e-4), MET4(1e-7)	amino acid metabolism (1.5e-30), nitrogen, sulfur and selenium metabolism (1.3e-13)
glycolysis regulation	38,78	Disulfide-reducing agent stress (1.6e-4), diamide (1.5e-3)	GCR1 (4.6e-3)	sugar, glucoside, polyol and carboxylate catabolism (3.3e-10), glycolysis and gluconeogenesis (3.1e-11)

*size:(the number of genes in the cluster, the number of conditions in the cluster)

## Conclusions

We have presented a rigorous hierarchical Bayes model for clustering microarray data in both the gene and the experimental condition directions. We used Gibbs sampling and Bayesian information criterion to identify biclusters as well as the total number of clusters. Using simulated datasets, we showed that the BBC algorithm outperformed other clustering methods especially when multiple clusters were present. Moreover, the BBC method performed the best for simulated data based on biochemistry models with realistic noise background. We also discussed the impact of normalization procedures on the clustering results, and found that both the interquartile range normalization and the smallest-quartile range normalization are robust for our BBC model. When applied to a well-known yeast microarray dataset, the BBC procedure discovered many biologically significant clusters, from which significant enrichments of gene functions, associated experimental conditions, and related TFBS enrichment were found.

Unlike many other biclustering methods, the BBC is completely model-based and does not need to fine tune any threshold parameters. Because it is a full Bayesian model, the BBC can handle missing data extremely easily, and can also incorporate likelihood-based criterion, such as AIC, BIC, maximum likelihood, Bayes factors, etc., for model evaluations and comparisons. In addition, the BBC model has the potential to be extended by incorporating other types of data, such as the promoter sequence information into the model.

## Methods

### Bayesian biclustering model

Consider a microarray dataset with *N* genes and *P* conditions (or samples), in which the expression value of the *i^th^* gene and *j^th^* condition is denoted as y*_ij_, i =* 1, 2, • • •, *N, j* = 1, 2, • • •, *P.* We assume that

$$y_{ij}=\sum_{k=1}^{K}((\mu_{k}+\alpha_{ik}+\beta_{jk}+\in_{ijk})\delta_{ik}\kappa_{jk})+e_{ij}(1-\sum_{k=1}^{K}\delta_{ik}\kappa_{jk}),$$

where *K* is the total number of clusters (unknown), μ*_k_* is the main effect of cluster *k*, and α*_ik_* and β*_jk_* are the effects of gene *i* and condition *j,* respectively, in cluster *k*, *ε_ijk_* is the noise term for cluster *k,* and *e_ij_* models the data points that do not belong to any cluster. Here δ*_ik_* and κ*_jk_* are binary variables: δ*_ik_* = 1 indicates that row (gene) *i* belongs to cluster *k*, and δ*_ik_* = 0 otherwise; similarly, κ*_jk_* = 1 indicates that condition (column) *j* is in cluster *k,* and κ*_jk_* = 0 otherwise.

When multiple biclusters are allowed, the original plaid model usually finds biclusters greatly overlapping with each other. This effect is quite artificial and is likely due to the nonidentifiability problem caused by the additive assumption made for overlapping clusters. We solve this problem by allowing biclusters to overlap only in one direction, either the gene or the condition direction, but not both. This results in two versions of the BBC model: non-overlapping gene biclustering and non-overlapping condition biclustering. In non-overlapping condition biclustering, a condition can be in one or none of the clusters, but a gene can be assigned to multiple clusters. Mathematically, this constraint can be written as $\sum_{k=1}^{K}\kappa_{jk}\leq 1$. In non-overlapping gene biclustering, a condition can be assigned into multiple clusters, while a gene can only be in no more than one cluster. This corresponds to $\sum_{k=1}^{K}\delta_{ik}\leq 1$. Note that in either of these two versions, different biclusters do not overlap. Without loss of generality, we focus our discussions on the non-overlapping gene biclustering in this paper. Thus the priors of the indicators κ and δ are set so that a condition can be in multiple clusters and a gene be in no more than one cluster, i.e.,

                                                                           *k_i__j_* ~ Bernoulli(*q_k_*)

$$P(\delta_{ik}=1,\delta_{il}=0,l\neq k)=p_{k}$$

$$P(\delta_{il}=0,l=1,2,\ldots K)=p_{0}=1-\sum_{k=1}^{K}p_{k},$$

where *p_k_* and q*_k_* are set to be constant. We tested different values for *p_k_* and *q_k_*, and found out that different values do not affect the results much. We used *q_k_* =0.1 and

$$p_{k}=\frac{1}{K+1}$$

for the yeast dataset.

We assume *a priori* that

$$\begin{array}{cc} \mu_{k} & ∼N(0,\sigma_{\mu k}^{2})\\ \alpha_{ik}|\delta_{ik}=1 & ∼N(0,\sigma_{\alpha k}^{2})\\ \beta_{jk}|\kappa_{jk}=1 & ∼N(0,\sigma_{\beta k}^{2})\\ \varepsilon_{ijk} & ∼N(0,\sigma_{\in k}^{2})\\ e_{ij} & ∼N(0,\sigma_{e}^{2}). \end{array}$$

The hyperpriors for the $\sigma_{\mu k}^{2},\sigma_{\alpha k}^{2},\sigma_{\beta k}^{2},\sigma_{\in k}^{2},\sigma_{e}^{2}$ are set to be inverse Gamma distributed.

In our model, an observation *y_ij_* can belong to either one or none of the biclusters. Thus, we can rewrite the probability distribution of *y_ij_* conditional on the cluster indicators. If *y_ij_* belongs to cluster *k*,(*k* = 1,2,… ,*K*), then

$$y_{ij}|\delta_{ik}=1,k_{jk}=1∼N(\mu_{k}+\alpha_{ik}+\beta_{jk},\sigma_{\in k}^{2}).$$

If *y_ij_* belongs to none of the clusters, then

$$y_{ij}|\delta_{ik}\kappa_{jk}=0\;for\;all\;k's∼N(0,\sigma_{e}^{2}).$$

With Gaussian zero-mean priors on the effects parameters, we get the marginal distribution of the *y_ij_* conditional on the indicators as:

Y|δ,κ∼N(0,∑),

where **Σ** is the covariance matrix of **Y**, and **Y** = (**Y_0_**, **Y_1_**, **Y_2_**, …, **Y_*K*_**)^*T*^ with **Y_*k*_** = {*y_ij_* : δ*_ik_*κ*_j__k_* = 1}, *k* ≥ 1, being the vector composed of the data points belonging to cluster *k*, and ***Y*_0_** being the vector of data points belonging to no cluster. More specifically, **Σ** is a sparse matrix of the form

$$\sum =\left(\begin{array}{cccc} \sigma_{e}^{2}I & 0 & \cdots & 0\\ 0 & \sum_{1} & \cdots & 0\\ \vdots & \vdots & \ddots & \vdots \\ 0 & 0 & \cdots & \sum_{K} \end{array}\right),$$

where **Σ*_k_*** = cov(**Y_*k*_**, **Y_*k*_**) is the covariance matrix of all data points belonging to cluster *k* whose entries are:

$$\begin{array}{c} \;\;\;\mathrm{cov}(y_{ij},y_{mn}|\delta_{ik}=\delta_{mk}=\kappa_{jk}=\kappa_{nk}=1)\\ =\{\begin{array}{ll} \sigma_{\mu k}^{2}+\sigma_{\alpha k}^{2}+\sigma_{\beta k}^{2}+\sigma_{\in k}^{2}, & if\;i=m\&j=n\\ \sigma_{\mu k}^{2}+\sigma_{\alpha k}^{2}, & if\;i=m\&j\neq n\\ \sigma_{\mu k}^{2}+\sigma_{\beta k}^{2}, & if\;i\neq m\&j=n\\ \sigma_{\mu k}^{2}, & if\;i\neq m\&j\neq n. \end{array} \end{array}$$

### Gibbs sampling for Bayesian biclustering

In order to make inference from the BBC model, we implement a Gibbs sampling method [27] to draw samples from the posterior distribution of the indicator variables, which can be derived by combining equation (8) with a prior distribution on the *δ* and *κ.* Initializing from a set of randomly assigned values of δ's and κ's, we sample the column (condition) indicators *κ* by calculating the following log-probability ratio:

$$\begin{array}{l} \log\frac{P(\kappa_{jk}=1|\kappa_{-j-k},\delta ..,\sigma_{\mu }^{2}.,\sigma_{\alpha }^{2}.,\sigma_{\beta }^{2}.,\sigma_{\in }^{2}.,\sigma_{e}^{2},Y)}{P(\kappa_{jk}=0|\kappa_{-j-k},\delta ..,\sigma_{\mu }^{2}.,\sigma_{\alpha }^{2}.,\sigma_{\beta }^{2}.,\sigma_{\in }^{2}.,\sigma_{e}^{2},Y)}\\ =\log\frac{P(Y|\kappa_{jk}=1|\kappa_{-j-k},\delta ..,\sigma_{\mu }^{2}.,\sigma_{\alpha }^{2}.,\sigma_{\beta }^{2}.,\sigma_{\in }^{2}.,\sigma_{e}^{2})P(\kappa_{ik}=1)}{P(Y|\kappa_{jk}=0|\kappa_{-j-k},\delta ..,\sigma_{\mu }^{2}.,\sigma_{\alpha }^{2}.,\sigma_{\beta }^{2}.,\sigma_{\in }^{2}.,\sigma_{e}^{2})P(\kappa_{ik}=0)}. \end{array}$$

Since each data point belongs to no more than one cluster, we can therefore divide data points into two sets given the current parameters except κ*_jk_*. The first set contains data points not in cluster *k*, i.e., **V**_1_ = {*y_il_* : δ*_ik_* = 0 or κ*_lk_* =0,*l* ≠ *j*}. The second set contains data points that are or can in cluster *k*, i.e., **V**_2_ = {*y_il_* : δ_*ik*_ = 1,κ_*lk*_ = 1,*l* ≠ *j*}U{*y_ij_* : δ_*ik*_ = 1}.

Two data points are independent if they belong to different clusters, therefore we can write the joint likelihood of **Y** as a product of the joint likelihood for data in **V**_1_ and **V**_2_, respectively. As a consequence, the log-posterior probability ratio can be simplified as

$$\log(\frac{P(V_{2}|\kappa_{jk}=1,\sigma_{\mu k}^{2},\sigma_{\alpha k}^{2},\sigma_{\beta k}^{2},\sigma_{\in k}^{2},\sigma_{e}^{2})P(\kappa_{ik}=1)}{P(V_{2}|\kappa_{ik}=0,\sigma_{\mu k}^{2},\sigma_{\alpha k}^{2},\sigma_{\beta k}^{2},\sigma_{\in k}^{2},\sigma_{e}^{2})P(\kappa_{ik}=0)}).$$

In order to calculate the likelihood term in the above ratio, we need to take the inverse and determinant of the covariance matrices for the vector **V**_2_ in both cases. The dimensions of these covariance matrices are huge in practice (in the order of thousands), so a brute force calculation would be expensive. Since the covariance matrices have the special structures as shown in equation (9), we can simplify the likelihood ratio term. The final simplified form only involves multiplications and additions of matrices with dimension *I_k_* × *I_k_*, where *I_k_* is the number of genes in cluster *k* given current parameters.

Similarly we can obtain the log- posterior probability ratio for gene indicators δ*_ik_*,

$$\log(\frac{P(\delta_{ik}=1|\delta_{-i-k},\kappa ..,\sigma_{\mu }^{2}.,\sigma_{\alpha }^{2}.,\sigma_{\beta }^{2}.,\sigma_{\in }^{2}.,\sigma_{e}^{2},Y)}{P(\delta_{ik}=0|\delta_{-i-k},\kappa ..,\sigma_{\mu }^{2}.,\sigma_{\alpha }^{2}.,\sigma_{\beta }^{2}.,\sigma_{\in }^{2}.,\sigma_{e}^{2},Y)}).$$

Since our model requires that $\sum_{k=0}^{K}\delta_{ik}\leq 1$, then the gene indicators δ*_ik_* are correlated. We thus need to calculate the log-posterior probability ratio for every δ*_ik_*, *k* = 1, 2, • • • *K*, and sample them jointly.

We also sample the effect parameters based on the indicators δ and κ. The gene effects α*_ik_* and condition effects β*_jk_* can serve as scores for genes and conditions in a cluster. Moreover the BBC model is very convenient and coherent in handling missing data in microarray datasets: just treat them as additional unknown variables and iteratively impute them in the Gibbs sampling iterations. Suppose at step *t*, we have sampled both the indicator variables and effects parameters, we can impute the missing data, say *y_ij_*, by sampling from distribution

$$\{\begin{array}{ll} N(\mu_{k}^{\left(t\right)}+\alpha_{ik}^{\left(t\right)}+\beta_{jk}^{\left(t\right)},\sigma_{\in k}^{2(t)}), & if\;\delta_{ik}^{\left(t\right)}\kappa_{jk}^{\left(t\right)}=1\\ N(0,\sigma_{e}^{2(t)}), & if\sum_{k=1}^{K}\delta_{ik}^{\left(t\right)}\kappa_{jk}^{\left(t\right)}=0 \end{array}$$

In the above procedure, we preset the value *K* for the total number of clusters. However, the information of *K* is not available in general. In practice, we search biclusters for a number of *K*'s and select the best *K* based on the Beyesian information criterion (BIC) [18].

An executable program for the BBC algorithm is available at 

## Competing interests

The authors declare that they have no competing interests.

## Authors' contributions

JG and JSL designed the models and simulation studies together. JG implemented the method and analyzed the data. Both authors contributed to the writing of the manuscript.
